# Using the Quantum Interface in Phenix to improve and automate metal coordination in macromolecular models

**DOI:** 10.1063/4.0000918

**Published:** 2025-10-27

**Authors:** Nigel W Moriarty

**Affiliations:** LBL, Berkeley, CA, USA

## Abstract

Quantum Mechanical methods provide geometries and energies of molecules using just the atomic and electronic positions. Being independent of the experimental data, they provide complementary information. Furthermore, allowing the experimental and QM methods to share information leads to better results.

The Quantum Interface (QI) in Phenix (Liebschner *et al.*, 2019) provides close integration with MOPAC (Moussa & Stewart, 2024) allowing the calculation of *in situ* restraints for drug candidates. Known as QM Restraints (QMR) (Liebschner *et al.*, 2023), this method will provide protein binding pocket-specific restraints during a refinement or in a stand-alone program.

Another QI procedure is a novel approach for predicting histidine protonation states using QM methods. Historically, metal coordination has been fraught.

The proposed method, Quantum Mechanical Ions (QMI), employs quantum mechanical calculations to predict metal coordination bond lengths and angles. The study demonstrates the effectiveness of QMI through comparisons with existing methods and validation using high-resolution protein structures.

Other applications of QI include histidine protonation (Moriarty *et al.*, 2024) and ligand strain energies, including energy comparisons of disordered ligands, all of which are being pursued.
